# The Attenuation Mechanism and Live Vaccine Potential of a Low-Virulence *Edwardsiella ictaluri* Strain Obtained by Rifampicin Passaging Culture

**DOI:** 10.4014/jmb.2210.10013

**Published:** 2022-12-09

**Authors:** Shuyi Wang, Jingwen Hao, Jicheng Yang, Qianqian Zhang, Aihua Li

**Affiliations:** 1 State Key Laboratory of Freshwater Ecology and Biotechnology, Institute of Hydrobiology, Chinese Academy of Sciences, Wuhan 430072, P.R. China; 2 University of Chinese Academy of Sciences, Beijing 100049, P.R. China; 3 Dalian Ocean University, Dalian 116023, P.R. China

**Keywords:** *Edwardsiella ictaluri*, rifampicin resistance, live vaccine, whole-genome sequencing

## Abstract

The rifampicin-resistant strain E9-302 of *Edwardsiella ictaluri* strain 669 (WT) was generated by continuous passage on BHI agar plates containing increasing concentrations of rifampicin. E9-302 was attenuated significantly by 119 times to zebrafish *Danio rerio* compared to WT in terms of the 50% lethal dose (LD_50_). Zebrafish vaccinated with E9-302 via intraperitoneal (IP) injection at a dose of 1 × 10^3^ CFU/fish had relative percentage survival (RPS) rates of 85.7% when challenged with wild-type *E. ictaluri* via IP 14 days post-vaccination (dpv). After 14 days of primary vaccination with E9-302 via immersion (IM) at a dose of 4 × 10^7^ CFU/ml, a booster IM vaccination with E9-302 at a dose of 2 × 10^7^ CFU/ml exhibited 65.2% RPS against challenge with wild-type *E. ictaluri* via IP 7 days later. These results indicated that the rifampicin-resistant attenuated strain E9-302 had potential as a live vaccine against *E. ictaluri* infection. A previously unreported amino acid site change at position 142 of the RNA polymerase (RNAP) β subunit encoded by the gene *rpoB* associated with rifampicin resistance was identified. Analysis of the whole-genome sequencing results revealed multiple missense mutations in the virulence-related genes *esrB* and *sspH2* in E9-302 compared with WT, and a 189 bp mismatch in one gene, whose coding product was highly homologous to glycosyltransferase family 39 protein. This study preliminarily explored the molecular mechanism underlying the virulence attenuation of rifampicin-resistant strain E9-302 and provided a new target for the subsequent study of the pathogenic mechanism of *E. ictaluri*.

## Introduction

Enteric septicemia of catfish (ESC) is a common disease of cultured channel catfish *Ictalurus punctatus*. At 22-28°C, ESC is acute septicemia with rapid development, and death usually occurs four days after infection [1]. Therefore, ESC inflicts considerable economic damage on the channel catfish aquaculture industry each year. The main bacterial pathogen causing ESC is *Edwardsiella ictaluri*, a facultative, intracellular gram-negative bacterium belonging to the Enterobacteriaceae and *Edwardsiella* genus, and a common bacterial pathogen in fish. *E. ictaluri* mainly infects Siluriformes, such as channel catfish *Ictalurus punctatus* [2, 3], Brazilian catfish *Pseudoplatystoma corruscans* [4], and yellow catfish *Pelteobagrus fulvidraco* [5, 6]. However, there have also been reports on mortality in non-catfish species, including Nile tilapia *Oreochromis niloticus* [7], rainbow trout *Oncorhynchus mykiss* [8], riverine ayu *Plecoglossus altivelis* [9], and zebrafish *Danio rerio* [10]. Especially in China, the frequent occurrence of bacterial diseases due mainly to *E. ictaluri* has led to serious economic losses in aquaculture and caused massive mortality of yellow catfish accompanied by splitting head (hole-in-the-head) disease [5], severe ascites, and enteric septicemia.

Although antimicrobial agents are still a common means of preventing and controlling outbreaks of bacterial fish diseases like edwardsiellosis, they have little effect as sick fish have a decreased feeding rate. In addition, the intensive use of antibiotics in aquaculture applies a selective pressure that creates reservoirs of drug-resistant bacteria [11] and pollutes the aquaculture environment. Therefore, immunoprophylaxis is the way forward for future drug development as new approaches are urgently needed to replace antibiotics to control explosive epidemic bacterial diseases. For this purpose, vaccination, which stimulates the immune system, can be an effective, alternative prevention strategy [12].

Studies have made it clear that mutagenesis using rifampicin is one of the most successful chemical mutagenesis strategies against gram-negative bacteria. Furthermore, the potential of live attenuated vaccines against fish pathogens based on rifampicin resistance has been demonstrated. Rifampicin is a broad-spectrum antibiotic drug belonging to the rifamycin family. Its action site is the β subunit of bacterial DNA-dependent RNA polymerase, which can inhibit bacterial DNA transcription at the initiation stage of transcription, so as to achieve sterilization [13]. In 1999, Klesius and Shoemaker successfully developed a live attenuated vaccine of *E. ictaluri*, which was created by multiple passages of a wild-type strain isolated from diseased channel catfish on increasing concentrations of rifampicin [1]. This vaccine was later patented [14]. In addition to *E. ictaluri*, the strategy based on the ability of rifampicin to induce rough mutants was successfully applied to *Flavobacterium columnare* [15] and *Brucella abortus* [16].

Antibiotic resistance due to chromosomal mutations is often accompanied by a fitness cost as antibiotic-resistant strains show reduced growth rates, decreased competitiveness, and attenuated virulence compared with the phenotype of antibiotic-sensitive parental wild strains [17]. Previous studies have shown that most rifampicin-resistant fish pathogens exhibit decreased virulence [18-20], but there has been no study on the correlation between fitness cost and rifampicin resistance. Some researchers believe that the fitness cost of resistance is the most important factor promoting the evolution of resistance in pathogen populations [21]. Hence, studying fitness cost can help in understanding the evolution of antibiotic resistance in bacteria.

In our study we sought to utilize this strategy on the highly pathogenic *E. ictaluri* isolated from diseased yellow catfish. The specific objectives were to: (i) generate rifampicin-resistant strains of *E. ictaluri* isolated from yellow catfish and screen out an attenuated strain using different kinds of fish, (ii) compare the phenotypic differences between the parent strain and the resistant strain to explore fitness cost of the resistant one, (iii) determine the virulence of the resistant strain in zebrafish, (iv) determine if immunization of zebrafish with the rifampicin-resistant strain confers a protective immune response against wild-type *E. ictaluri* challenge, and (v) investigate the possible mechanism of attenuation and explore potential virulence factors of *E. ictaluri* based on whole-genome sequencing analysis.

## Materials and Methods

### Bacterial Strains and Culture Conditions

Table S1 shows the name and origin of all strains used in our study, which were confirmed to be *E. ictaluri* isolates by sequencing. Bacteria strains were stored at -80°C as glycerol stocks and cultured on Brain Heart Infusion (BHI) agar plates or BHI broth with shaking (200 ×*g*) at 28°C.

### Diversity Analysis of *E. ictaluri*

Ten housekeeping genes (*adk*, *atpD*, *dnaJ*, *gapA*, *glnA*, *Y-hsp60*, *phoR*, *pyrG*, *rpoA*, *tuf*) were selected as targets for PCR assays. The housekeeping genes and the primers used for multilocus sequence typing (MLST) according to the public databases for MLST of *Edwardsiella* (*Edwardsiella* spp. | PubMLST) are shown in Table S2 (slightly modified). The above *E. ictaluri* isolates from different origins (Table S1) were expanded to the logarithmic growth phase. Genomic DNA was extracted from logarithmic-phase bacterial cultures using a Bacterial DNA Kit (China) according to the manufacturer’s instructions. The concentration and quality of extracted genomic DNA was measured using a NanoDrop 1000 spectrophotometer (Thermo Fisher Scientific, Inc., USA), and DNA was stored at -20°C until used as a template for amplification.

PCR amplification was carried out in a 50 μl reaction mixture containing 19 μl sterile ultrapure water, 2 μl of each pair of specific upstream and downstream primers, 2 μl of template DNA, and 25 μl of 2×Es Taq MasterMix (containing Es Taq DNA Polymerase, 3 mM MgCl_2_ and 400 μM each dNTP) (Dye) (CWbio, China). The optimized amplification program used in a thermal cycler (iCycler, Bio-Rad Laboratories, USA) was initial denaturation at 94°C for 2 min, 35 cycles containing denaturation at 94°C for 30 s, annealing at 55-65°C for 30 s, and elongation at 72°C for 30 s followed by a final extension of 72°C for 2 min. The PCR products were detected by 1% agarose gel electrophoresis and purified using a FastPure Gel DNA Extraction Mini Kit (Vazyme, China). The purified products were ligated into vector pMD-18T and transformed into competent *Escherichia coli* DH5α cells. Positive clones were selected by coating ampicillin-resistant plates and sent out to Tsingke Biotechnology Co., Ltd., China, for bidirectional sequencing.

The sequences of ten housekeeping genes of each strain were submitted to the database, and the allelic numbers with the best match for each gene were obtained. The sequence type (ST) was obtained according to standard procedure. In addition, the allelic tandem sequences of *E. ictaluri* isolates with known STs (Table S3) were downloaded from the database. MEGA v.11.0 software was used to align the tandem sequences and construct the dendrogram, which was used to analyze the genetic and evolutionary relationships between the strains stored in our laboratory and the isolates from different hosts in different years in other regions. The best model was selected from 24 replacement models according to the Bayesian Information Criterion (BIC), and the Maximum Likelihood method of the Tamura 3-parameter model was used to construct the phylogenetic tree. The bootstrap test was used (1000 replicates) to test the quality of the tree.

### Generation of Rifampicin-Resistant Strains by Subculture

Rifampicin (analytical purity, AR; Kehbio, China) was dissolved in methanol (AR) to a concentration of 5 mg/ml, which was sterilized with a sterile 0.22 μm syringe filter and stored at -20°C until use. The generation of rifampicin-resistant strains was based on an existing procedure [1] with minor modifications. Two virulent strains of *E. ictaluri* isolated from diseased yellow catfish, strain 668 and strain 669, were used as the parent strains to induce the development of rifampicin-resistant strains. The minimal inhibitory concentration (MIC) of rifampicin against strain 668 and strain 669 was 2 μg/ml, and the initial concentration of rifampicin that allowed the growth of *E. ictaluri* was 1.5 μg/ml. After 20 passages (the concentrations of rifampicin in BHI agar plates were 1.5, 3, 6, 8, 10, 12, 16, 20, 25, 30, 50, 70, 90, 120, 150, 180, 210, 240, 270, and 300 μg/ml, respectively) of *E. ictaluri* strain 668 or strain 669 in BHI agar containing progressive concentrations of rifampicin, and the rifampicin-resistant strains were able to grow in BHI agar containing 300 μg/ml. Strains were identified by sequencing and stored at -80°C until use.

### Screening of an Attenuated Rifampicin-Resistant Strain

**Fish**

Blue gourami *Trichogaster trichopterus* and zebrafish were purchased from a local aquarium, and Nile tilapia were purchased from Yingshan Tilapia Breeding Base. Fish were temporarily raised for 2 weeks before the experimental operation, and 5% of fish were randomly selected for detection of *E. ictaluri* in the liver, kidney, and spleen. Only fish without detection of *E. ictaluri* could be used in subsequent experiments. The keeping and experimentation temperature regarding the fish was set at 26 ± 2°C.

### Primary Screening of Attenuated Rifampicin-Resistant Strains

Blue gourami have often been used as an experimental model to study the mechanisms of bacterial pathogenesis and evaluate the efficacy of vaccines in previous studies [12, 22, 23]. Blue gourami (4 cm average length, 20 fish per group) were IP injected with the same high dose (5 × 10^7^ CFU/fish) of sterile phosphate-buffered saline (PBS)-resuspended strain 668 and strain 669 and their corresponding rifampicin-resistant strains, while the control group was IP injected with the same volume of sterile PBS. The strains with fewer deaths were selected as the attenuated rifampicin-resistant strains obtained from the primary screening.

### Secondary Screening of Attenuated Rifampicin-Resistant Strains

Nile tilapia (6 cm average length, 20 fish per group) were IP injected with the same high dose (5 × 10^7^ CFU/fish) of sterile PBS-resuspended strains obtained from primary screening and their corresponding wild-type strains, while the control group was IP injected with the same volume of sterile PBS. The strain with the fewest deaths was selected as the final attenuated rifampicin-resistant strain and named E9-302.

### Fitness Costs due to Resistant Mutations

**Comparison of *rpoB* Sequence**

Since rifampicin inhibits bacterial RNA synthesis by specifically targeting a small but highly conserved pocket in the RNA polymerase (RNAP) subunit encoded by *rpoB* [24], 95% of rifampicin resistance mutations are present in the *rpoB* [25]. Difference in *rpoB* sequence between wild-type strain and resistant strain was the most direct fitness cost associated with rifampicin resistance [26]. Comparison of *rpoB* was completed by subsequent whole-genome sequencing.

### Cell Proliferation Profile of E9-302 Compared to WT

A slower growth rate has been reported as a fitness cost of antibiotic-resistant bacteria [27, 28]. Cell proliferation assays were performed according to published procedures [29] with slight modifications. Briefly, both WT and E9-302 were cultured overnight in BHI at 28°C, 200 rpm. The optical density (OD) of each strain at 570 nm was determined and adjusted to OD_570 nm_ = 1. Seven dilutions of two bacterial solutions (1:10, 1:20, 1:40, 1:80, 1:160, 1:320, 1:640) and a blank control of fresh BHI medium were set in sterile 96-well microtiter plates in triplicates per strain and incubated at 28°C after adding the bacterial solution in proportion to each dilution gradient. The OD of the 96-well plate measured at 570 nm soon after the addition of the bacterial solution was considered as the initial OD value for 0 h and the OD_570 nm_ was measured every hour. Relative increased OD value was calculated using the following formula: Increased OD_570 nm_ value = OD_570 nm_ value after incubation - OD_570 nm_ value at 0 h of the incubation. The increased OD_570 nm_ value after incubation was then plotted against incubation time.

### In Vitro Competition Assay

The cost of drug-resistant mutations was reported to be determined by direct competition against drug-sensitive parental strains [30]. The in vitro competition assays were performed according to published procedures [30] with slight modifications: After adjusting the OD of WT and E9-302 at 600 nm to OD_600 nm_ = 1, an equal volume of each strain was mixed to form 1 ml of bacterial broth and transferred to antibiotic-free BHI medium at a ratio of 1:100 for incubation at 28°C for 14 h. Then, 1 ml of the culture was centrifuged at 2380 ×*g* for 5 min and resuspended in sterile PBS. The bacterial suspension was coated on antibiotic-free BHI agar plates and the number of colonies was counted as the total number of bacterial cells. The number of colonies obtained by counting the same volume of bacterial suspension coated on rifampicin-resistant BHI agar plates was taken as the number of E9-302 colonies, and the number of WT colonies = total number of bacterial cells - the number of E9-302 colonies. The assays were performed in triplicate with three independent cultures. Serial dilutions of each culture were plated and counted three times to take the mean value. The difference in fitness cost between two competing strains at time *t* was calculated using the following formula:

$$S_{t}=\ln\left[{\left(\frac{r_{t}/S_{t}}{r_{t-1}/S_{t-1}}\right)}^{\frac{1}{8}}\right]$$ (1)

Where *r_t_* and *S_t_* represent the absolute number of rifampicin-resistant strain E9-302 and rifampicin-sensitive strain WT at time t, respectively, *r_t−1_* and *S_t−1_* represent the absolute number of rifampicin-resistant strain E9-302 and rifampicin-sensitive strain WT at the previous time point, respectively. *S_t_* is called the selection coefficient at time t. *r_t_*/*r_t−1_* and *S_t_*/*S_t−1_* represent the growth rate of rifampicin-resistant strain E9-302 and rifampicin-sensitive strain WT, respectively. Hence, S can represent the natural logarithm of the quotient of the growth rate of competing strains.

According to the research, *S* is positive if rifampicin resistance increases bacterial fitness, *S* is equal to 0 if there is no difference in fitness between two competing strains, and *S* is negative if rifampicin resistance incurs fitness cost.

### Virulence Determined in Zebrafish

Zebrafish were used to determine the 50% lethal doses (LD_50_) of the WT and E9-302. Zebrafish (length, 3 ± 0.2 cm) were fed a basal diet and temporarily kept for 7 days before the experiment. Groups of 20 fish were IP injected using microinjectors (Hamilton, Switzerland) of a 50 μl range with 5 μl of WT and E9-302 at different concentration gradients (10^2^ ~ 10^7^ CFU/fish) with three parallel groups. The blank control was IP injected with the same volume of sterile PBS. Survival of zebrafish was observed and recorded for 7 days, and LD_50_ was estimated with the Spearman-Kärber method [31] modified from Dias [32].

### Vaccination Trials in Zebrafish

Zebrafish (length, 3 ± 0.2 cm) were divided into 12 parallel experimental groups of 20 fish each. In groups 1-3 and groups 4-6, fish were inoculated with E9-302 or PBS via IP injection, respectively. In groups 7-9 and groups 10-12, fish were inoculated with E9-302 or PBS via immersion (IM). For the IP injection group, the inoculation dose was 1 × 10^3^ CFU/fish, that was the experimental fish were IP injected using microinjectors as mentioned before with 5 μl of 2 × 10^5^ CFU/ml E9-302, while the control fish were IP injected with the same volume of sterile PBS. At 14 days post-vaccination, the vaccinated fish from each group (30 fish) were challenged via IP injection with 1 × 10^4^ CFU/fish of parental *E. ictaluri* WT. For the IM group, zebrafish were immersed in 4 L of sterile PBS resuspension of E9-302 with a concentration of about 4 × 10^7^ CFU/ml for 40 min, while the control fish were immersed in sterile PBS with the same volume for the same length of time, and continuous aeration was maintained in all groups. A booster vaccination via IM was conducted upon 14 days after primary IM. The experimental settings were consistent with the primary IM except for the IM concentration of 2 × 10^7^ CFU/ml. Thirty fish from each treatment group (groups 1-3, groups 4-6, groups 7-9, and groups 10-12) were randomly selected and challenged with 1 × 10^4^ CFU/fish of parental *E. ictaluri* WT upon 7 days after booster vaccination. The mortality of each group was recorded for both IP infection group and IM group after challenge, and the results were expressed as relative percentage survival (RPS) as previously described [33]. RPS was computed according to the following formula:

RPS = [1-(vaccinated mortality/control mortality)]×100%. (2)

### Whole-Genome Sequencing

The bacterial whole-genome sequencings of WT and E9-302 were completed by a combination of next-generation sequencing and SMRT, *i.e.*, Illumina Hiseq sequencing combined with single-molecule PacBio sequencing at the Majorbio Biotech Co., Ltd. (China). Briefly, next-generation sequencing started with the purification of genomic DNA, genomic DNA fragmentation using Covaris to construct a genomic sequencing library, and Bridge PCR followed by IlluminaHiseq sequencing. As for SMRT, the purified genomic DNA was fragmented using the G-tube method to construct the SMRT Bell library, which was then annealed and bound to the bottom polymerase of zero-mode waveguides (ZMW). The libraries were quantified by Qubit and the inserted fragment size was measured by Agilent 2100, and finally sequenced using the PacBio platform. The differences in genes related to virulence between WT and E9-302 were compared and analyzed to examine the underlying genomic basis of attenuation.

## Results

### Diversity Analysis of *E. ictaluri*

To produce an MLST assay we used ten housekeeping genes (Table S2), which were bidirectionally sequenced and then submitted to the database to obtain the allelic numbers with the best match for each gene. The sequence type (ST) of each strain was computed according to the standard procedure provided by the database (Table 1). The results showed that the STs of *E. ictaluri* isolated from different origins were consistent, as all were ST26, showing the high degree of genetic homogeneity among *E. ictaluri* in China. Accordingly, the results also made it possible to develop widely applicable vaccines against edwardsiellosis in China.

**Fig. 1:**
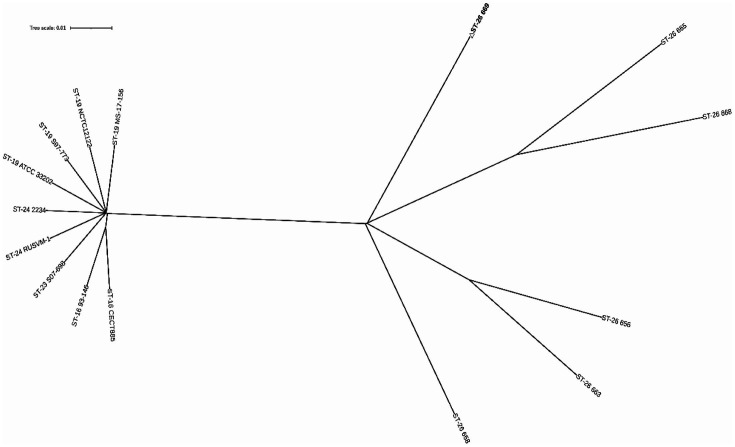
Phylogenetic tree based on tandem sequence (*adk*- *atpD*- *dnaJ*- *gapA*- *glnA*- *Y-hsp60*- *phoR*- *pyrGrpoA*- *tuf*) showing relationships of different STs of fifteen *E. ictaluri* isolates in this study.

The phylogenetic tree of fifteen *E. ictaluri* isolates (Tables S1 and S3) was constructed using MEGA v.11.0 software based on tandem sequence (*adk*- *atpD*- *dnaJ*- *gapA*- *glnA*- *Y-hsp60*- *phoR*- *pyrG*- *rpoA*- *tuf*) with the Maximum Likelihood method and Tamura 3-parameter model (Fig. 1). The genetic correlations among *E. ictaluri* isolates investigated in this study showed that the isolates were largely clustered according to sequence type (ST). The sequence type of the *E. ictaluri* prevalent in China was mainly ST26, and isolates in China were distantly related to those from other countries and regions.

### Screening and Obtaining of an Attenuated Vaccine Candidate

**Fig. 2:**
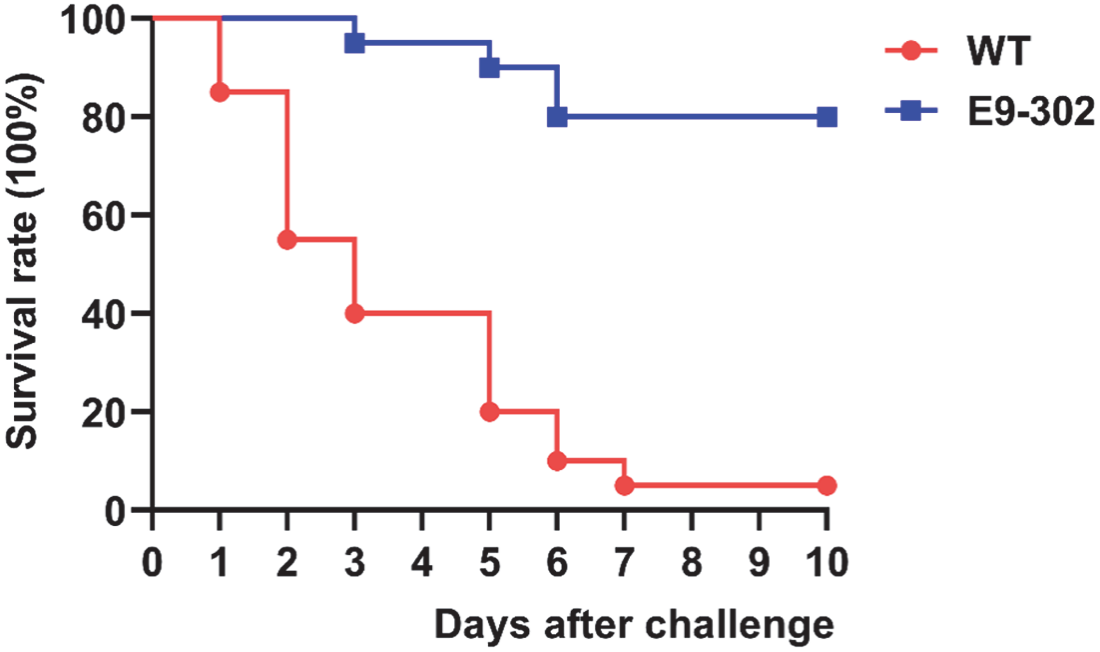
Survival curve of blue gourami IP injected with equal high dose (5 × 10^7^ CFU/fish) of WT and E9-302.

**Fig. 3:**
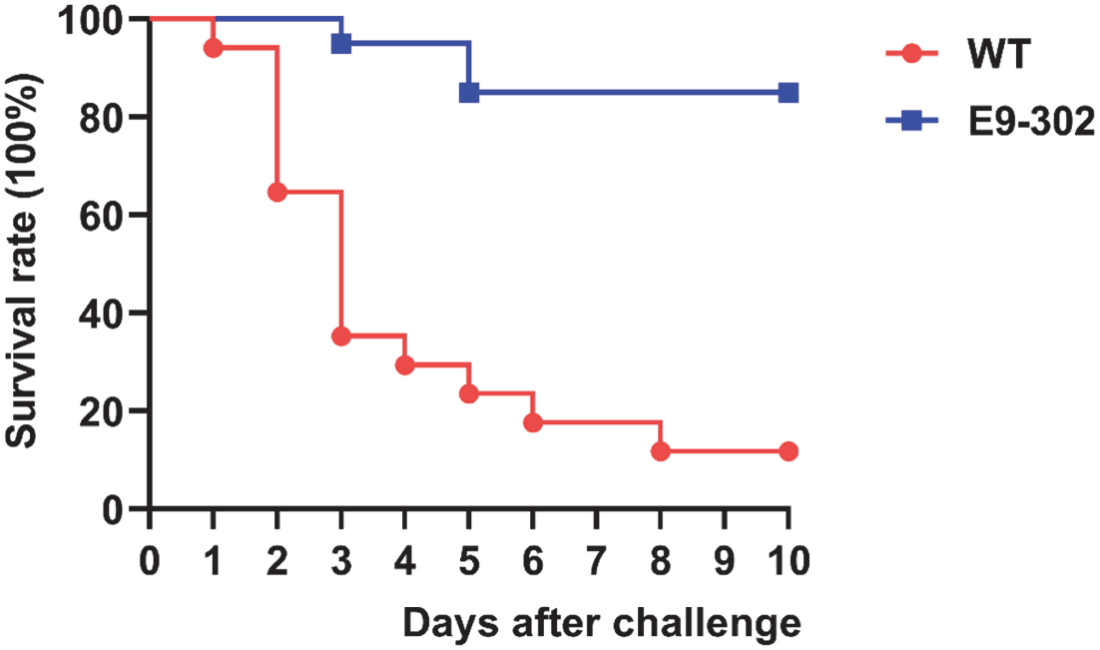
Survival curve of Nile tilapia IP injected with equal high dose (5 × 10^7^ CFU/fish) of WT and E9-302.

The strain E9-302 was a rifampicin-resistant mutant, which was produced by the highly pathogenic *E. ictaluri* strain 669 (WT) isolated from yellow catfish by continuous passages on BHI agar plates containing increasing concentrations of rifampicin. Blue gourami and Nile tilapia as the experimental subjects were IP injected with the same dose of high-concentration sterile PBS resuspension of each alternative strain to perform virulence screening. After two rounds of screening, we selected the strain with the fewest deaths as the attenuated rifampicin-resistant vaccine candidate and named it E9-302. Figs. 2 and 3 showed survival curves of WT and E9-302 in two rounds of virulence screening, respectively.

### Fitness Cost Analysis of E9-302

#### Sequencing Results of *rpoB*

The *rpoB* sequences of WT and E9-302 were both 4,029 bp in length, coding for 1,342 amino acids. Sequence alignment between *rpoB* of WT and that of E9-302 revealed the three missense mutations (Table 2).

#### Cell Proliferation Profile of E9-302 Compared to WT

**Fig. 4:**
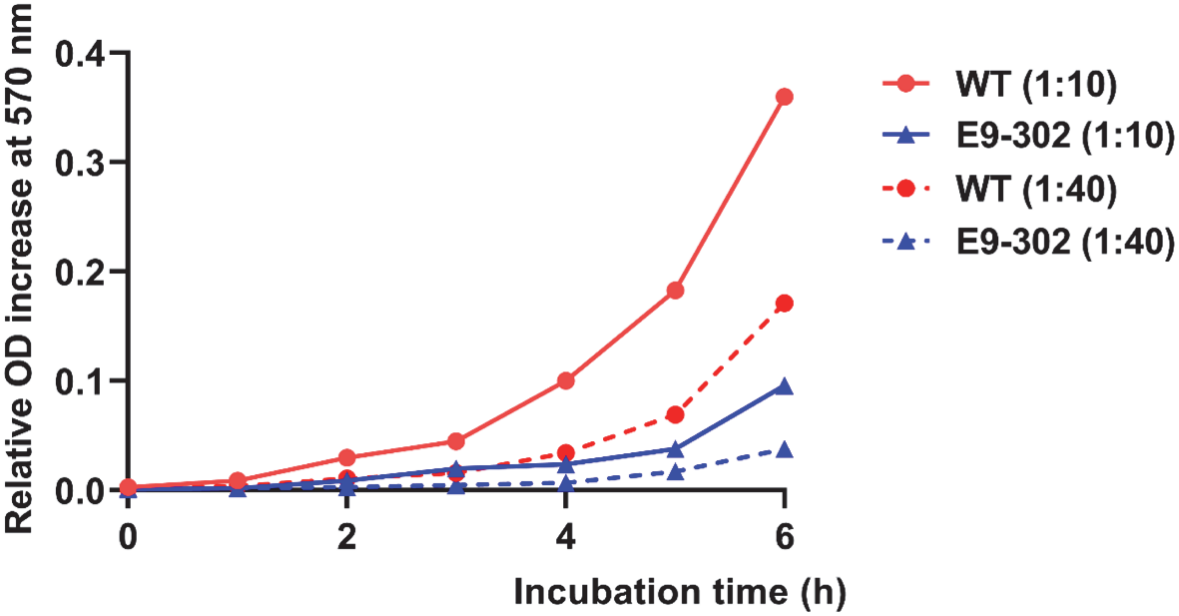
Cell proliferation rate of *E. ictaluri* E9-302 compared to that of *E. ictaluri* WT.

When the initial amount of bacteria was either at 1:10 or 1:40, the relative increase of the OD_570 nm_ values of WT from 0 h to 6 h was higher than that of E9-302, but not significant (Fig. 4). Similar patterns were observed as well when the beginning amount of both bacteria in each well was at other dilutions (Fig. S1).

#### In Vitro Competitiveness of E9-302 Compared to WT

The initial bacteria amount of WT and E9-302, the bacteria amount after 14 h of incubation, and the S value were shown in Table 3. The S value, which can be interpreted as the natural logarithm of the quotient of growth rates of competing strains, was used to judge fitness differences. Data were shown as the mean of three independent parallel experiments. As shown in Table 3, the fitness difference *S* < 0 indicated that resistance to rifampicin reduced the fitness of bacteria.

#### Assessment of Virulence

Zebrafish have been developed as a powerful laboratory model to study the pathogenesis of bacterial fish pathogens [34-36], including *E. ictaluri* [37-39]. As shown in Table 4, *E. ictaluri* E9-302 was attenuated with an LD_50_ of 1.04 × 10^5^ CFU/fish, a 119-fold increase over the parental strain WT, indicating that the virulence of E9-302 was significantly weakened. The dying zebrafish exhibited clinical symptoms associated with edwardsiellosis such as body spinning, head up and tail down in the water, blood spots on the body surface, and *E. ictaluri* was detected in internal organs.

#### Immunoprotective Effects of E9-302 in Zebrafish

The zebrafish were vaccinated with E9-302 via IP injection or IM and challenged with 1 × 10^4^ CFU/fish of wild-type *E. ictaluri* strain WT at 14 days after injection or 7 days after booster IM. When zebrafish vaccinated E9-302 via IP injection were challenged with WT at 14 dpv, the RPS of vaccinated fish was 85.7% (Table 5), and when zebrafish vaccinated E9-302 via IM with booster IM were challenged with WT at 7 days after booster IM, the RPS of vaccinated fish was 65.2% (Table 5), indicating E9-302 provided protection against the parental *E. ictaluri* strain WT, and thereby showing its potential as a vaccine against edwardsiellosis.

#### Genomic Analysis of E9-302 Compared to WT

The whole-genome sequencing comparison showed that in addition to the differences in the gene *rpoB* as mentioned before, the genes *esrB*, *sspH2*, and a gene of unknown function (named Gt39-like in this paper), were also mutated. There was one missense mutation in *esrB*, a 27 bp sequence insertion and multiple missense mutations in *sspH2*, and a 189 bp mismatch in gene Gt39-like (Table 6). The gene *esrB* encodes EsrB, a response regulatory (RR) element in the two-component EsrA-EsrB system, and the gene *sspH2* encodes the E3 ubiquitin ligase SspH2.

As for the gene Gt39-like with unknown function in WT, BLAST alignment showed that the amino acid sequence (488 AA) was highly homologous with the amino acid sequences of glycosyltransferase family 39 protein (GT39) of *Salmonella enterica* (NCBI Accession: WP_183051533.1), *Yokenella regensburgei* (NCBI accession: WP_040903585.1), and *Klebsiella michiganensis* (NCBI Accession: WP_004131974.1).

## Discussion

Multilocus sequence typing (MLST) is a molecular typing method for bacteria based on nucleic acid sequence sequencing. Since it was first established and applied in 1998 for *Neisseria meningitides* [40], MLST has come to be widely used in other pathogens or environmental microorganisms due to its high sensitivity and specificity, and is now recognized as a more accurate and critical means to detect bacterial epidemiology, study evolution, and assess genetic diversity [41]. The typing results of the *E. ictaluri* isolates preserved in our laboratory were consistent, with all being classified as ST26. In addition, the plasmid profiles of *E. ictaluri* isolates from different provinces and cities in China stored in our laboratory all showed two plasmids (data not shown), which was consistent with the conclusion of Liu *et al*. [5]. These findings indicated a high degree of genetic homogeneity of *E. ictaluri* isolates in China and offered the possibility to develop efficient vaccines against edwardsiellosis with wide applicability in China. In this study, the strategy for exploiting rifampicin-resistant attenuated pathogens was applied to the highly pathogenic *E. ictaluri* isolated from diseased yellow catfish. After multiple rounds of screening, an attenuated rifampicin-resistant strain of *E. ictaluri* named E9-302 was successfully obtained, which was 150 times more resistant to rifampicin than its parental strain (WT). Meanwhile, the commercial ESC vaccine Aquavac-ESC (RE-33) was only 6.4-fold resistant to rifampicin compared to its parent strain EILO [1]. Virulence assessment suggested a significant decrease in virulence with a 119-fold reduction in the 50% lethal dose (LD_50_) of E9-302 to zebrafish compared to highly pathogenic WT, which was in agreement with previous findings showing reduced virulence [18] of rifampicin-resistant pathogenic strains. The attenuated strain E9-302, inoculated into zebrafish in different ways, had a protective effect against challenge with the parental wild-type *E. ictaluri* strain WT, producing 85.7% (by IP route) and 65.2% (by IM plus booster IM route) RPS respectively, which was good evidence of the potential of E9-302 as a live attenuated vaccine against edwardsiellosis. Klesius *et al*. immersed the channel catfish in 1 × 10^5^ CFU/ml of RE-33 for 2 min, and the fish were challenged by immersion in 1 to 2 × 10^7^ CFU/ml of the parental strain (EILO) for 60 min upon 14 days post-vaccination, and the RPS of vaccinated fish was 51.7% [1]. However, since the fish species, immunization concentration, immunization time, and challenge mode were different, whether E9-302 will also provide higher protection to the same size of channel catfish by the same route merits further study.

Mutations in antibiotic resistance are often accompanied by virulence reduction and other fitness costs [33, 42, 43]. In clinical medicine, the fitness cost of antibiotic resistance is the basis for studying methods to limit the spread of drug-resistant mutants, which is helpful in researching the evolution of bacterial antibiotic resistance [21]. At present, no investigation is being undertaken on the fitness cost of rifampicin-resistant fish pathogens, and this is the first study on the correlation between fitness cost and rifampicin resistance. In addition to the decreased virulence, our results also showed that the cell proliferation rate of E9-302 was lower than that of WT (Fig. 4). Studies have shown that one of the fitness costs of antibiotic-resistant strains is reduced growth rate [27, 28], and this conclusion is also supported by the fact that the growth rate of rifampicin-resistant *Streptococcus iniae* strain D3-r25 was significantly lower than that of its parent *S. iniae* strain D-WT [44]; and the cell proliferation rate of novobiocin-resistant *S. iniae* strain ISNO was apparently lower than that of its virulent parental *S. iniae* strain ISET0901 [45]. The difference in fitness S calculated from in vitro competition experiments is less than zero, also indicating that resistance to rifampicin incurs a fitness cost. As the broad-spectrum antibiotic rifampicin specifically targets RNAP, changes in the gene encoding the drug target enzyme RpoB represent the most direct fitness cost of rifampicin resistance. The differences in the gene *rpoB* of rifampicin-resistant and sensitive strains of both *Mycobacterium kansasii* (three mutations in codons 513, 526, and 531) [46] and *Escherichia coli* (seventeen mutations in three distinct clusters in the center of the gene *rpoB*) [47] also validated this view. Here, we compared the *rpoB* sequences of E9-302 and WT on the basis of whole-genome sequencing and found a total of three missense mutations (Table 2). Among them, the 509th and 511th amino acid sites of RNAP β subunit encoded by the gene *rpoB* were in rifampicin resistance determination region (RRDR) cluster II, and the 511th amino acid residue was one of the amino acid residues directly associated with rifampicin binding, verifying the direct fitness cost from rifampicin resistance. Additionally, the 142nd amino acid with missense mutation has not been reported in the literature. The above results indicated that, in the absence of antibiotics, E9-302 was in an inferior position compared with WT in terms of growth rate and competitiveness. Changes in the gene *rpoB* on chromosome did confer fitness costs on antibiotic-resistant E9-302, suggesting that the growth and transmission of E9-302 might likewise be limited in the natural environment.

One of the common approaches to developing live attenuated vaccines is to obtain less virulent or even non-virulent mutants from highly pathogenic wild-type strains through multiple rounds of gradient increasing rifampicin concentration. As mentioned earlier, many successful cases have demonstrated that this approach is effective and feasible for generating attenuated mutants, but the mechanism leading to attenuation of virulence is still unclear. There may be various attenuation mechanisms of the same antibiotic on different bacteria. Here, we conducted whole-genome sequencing for WT and E9-302, aiming to preliminarily explore the molecular mechanism of rifampicin-attenuated *E. ictaluri*.

In our study, compared with WT, whole-genome sequencing analysis revealed a missense mutation in the E9-302 *esrB* gene, which encodes EsrB, a response regulatory element of the EsrA-EsrB two-component system. EsrA-EsrB of *Edwardsiella* is homologous to SpiR-SsrB/SsrA-SsrB of *Salmonella*. In *Salmonella*, the two-component system SsrA-SsrB is encoded by Pathogenicity Island 2 (SPI-2) [48]. From the homology of *Salmonella* and *Edwardsiella*, it can be inferred that EsrA-EsrB of *Edwardsiella* also plays a regulatory role in the pathogenic process. At present, the regulation of major virulence factors by the two-component system EsrA-EsrB of *Edwardsiella* has been studied; for instance, the gene *esrB* regulates the secretion of the essential secretory proteins EseB, EseC, and EseD of T3SS [49], and the effector protein EvpP of T6SS in *E. tarda* [50] also strongly induces the expression of T3SS/T6SS in *E. piscicida* [51]. In summary, the EsrA-EsrB two-component system has regulatory effect on both T3SS and T6SS of the host “weapons” of *Edwardsiella* pathogen infection. It was speculated that the function of the gene *esrB* with a missense amino acid mutation in this study might be affected, which then led to a drop in virulence. The regulatory effect of the gene *esrB* on the secretion system and other virulence factors in *E. ictaluri* needs to be further verified by gene knockout and site-directed mutagenesis.

Ubiquitination is an integral signal for the host immune response to pathogenic pathogens [52, 53]. It is precisely because of its crucial role that the host ubiquitination pathway becomes a target of pathogen hijacking. Pathogens secrete effector proteins that mimic eukaryotic ubiquitin ligase E3 or encode novel E3 ligases (NELs) that are different in sequence and structure from any eukaryotic ubiquitin ligase E3 [54]. They can also encode deubiquitinating enzymes to manipulate the host ubiquitination process [55], thereby facilitating bacterial entry into the host cell, setting up a replication environment and accelerating the infection process. In this study, the gene *sspH2* in E9-302 had multiple missense mutations (Table 6), which likely affected its normal function. The *E. ictaluri* gene *sspH2* is homologous to the gene *sspH2* encoding protein SspH2 of the E3 ubiquitin ligase family in *Salmonella*. In *Salmonella*, the T3SS2 encoded by pathogenicity island 2 (SPI-2) is activated following invasion of host cells, which is essential for intracellular survival and proliferation [56]. SspH1, SspH2, and SlrP, the important effector proteins of T3SS2, are three members of the *Salmonella* NELs, all of which contribute to virulence in pathogen infection [57-59]. It is worth noting that SspH2, encoded by gene *sspH2*, could assist pathogens to invade host cells and advance themselves intracellularly to disrupt host immune responses [60-67]. Based on the protein structure and homology, it is hypothesized that *E. ictaluri* SspH2 may also have a regulatory or even disruptive effect on the host immune response, and the conjecture will be subsequently verified by a combination of multiple techniques including deletion strain construction, as well as the combination of dual luciferase reporter genes and immunoprecipitation.

Glycosylation modifications of bacterial proteins have a major impact in many of their life activities including adhesion, colonization, pathogenicity and immune escape [68-70]. In another gene named Gt39-like with differences in this study, E9-302 was missing a base compared with WT, resulting in a mismatch of the subsequent 189 bp sequence (Table 6). Alignment revealed that the amino acid sequences encoded by this gene were highly homologous to that of GT39 of *S. enterica*, *Y. regensburgei*, and *K. michiganensis*. It was found through comparison with the CAZy database that the protein encoded by the gene Gt39-like belonged to dolichol-phosphate-mannose-protein mannosyltransferase (PMT), a relatively conserved GT39 protein in all organisms. PMT acts as a catalyst for the initial step of protein mannosylation by catalyzing the transfer of mannosyl residues to serine or threonine residues [71], and plays an indispensable part in protein mannosylation. Studies have shown that PMT plays an instrumental role in maintaining the virulence of *Mycobacterium tuberculosis* [72] and contributes to the adherence and invasive ability of *Campylobacter jejuni* [73]. Up to the present, protein mannosylation in prokaryotes has been poorly studied. The product encoded by the gene named Gt39-like may also have a part in virulence and adhesion of *E. ictaluri*, which needs to be verified by subsequent knockout.

In this study, the strategy for exploiting rifampicin-resistant attenuated pathogens was applied to the highly pathogenic *E. ictaluri* isolated from diseased yellow catfish. An attenuated strain E9-302 with a 119-fold decrease in LD_50_ in virulence to zebrafish in comparison to the parental strain WT was obtained. The zebrafish inoculated with strain E9-302 in different ways showed protection against challenge with the parental wild-type *E. ictaluri* strain WT, producing 85.7% (by IP route) and 65.2% (by IM plus booster IM route) RPS respectively, suggesting the potential of E9-302 as a live attenuated vaccine against edwardsiellosis. The virulence assay combined with the comparison of cell proliferation rate and in vitro competition ability of the parental strain WT and E9-302 proved for the first time that the acquisition of rifampicin resistance gave fish pathogens fitness costs in terms of reduced pathogenicity, slower cell proliferation, and decreased competitiveness. Furthermore, the differences in the gene *rpoB* sequences between the two on the basis of whole-genome sequencing emphasized the generation of fitness cost again. Additionally, whole-genome sequencing revealed that mutations in the gene *esrB*, the gene *sspH2* encoding E3 ubiquitin ligase SspH2, and the gene named Gt39-like encoding the glycosyltransferase family 39 protein, were likely to be associated with virulence attenuation in E9-302. For these reasons, this work provides new ideas and targets for subsequent studies on the pathogenesis of *E. ictaluri*.

## Supplemental Materials

Supplementary data for this paper are available on-line only at http://jmb.or.kr.

## Figures and Tables

**Fig. 5:**
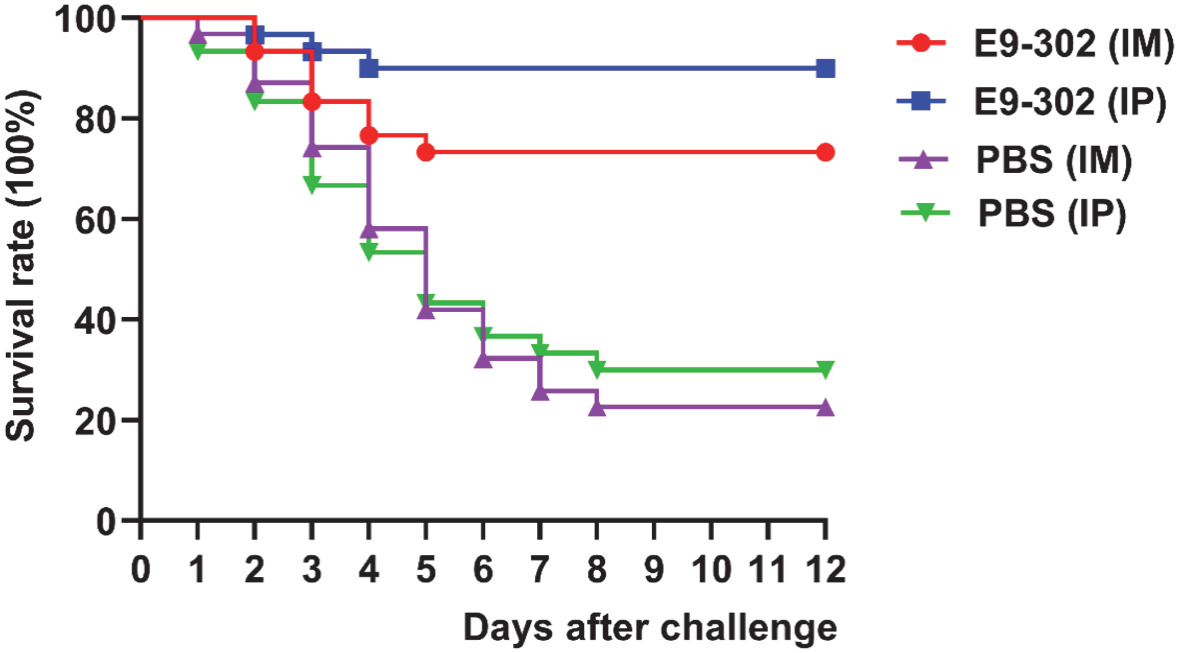
Survival curves of the IP immunized group, the IM immunized group, and respective control groups after the strain WT challenge.

**Table 1:** STs of *E. ictaluri* isolates in our study.

Strain	Allelic numbers with the best match for each gene										ST

	*adk*	*atpD*	*dnaJ*	*gapA*	*glnA*	*Y-hsp60*	*phoR*	*pyrG*	*rpoA*	*tuf*	
656	9	10	10	10	8	2	12	12	6	7	26
658	9	10	10	10	8	11	12	12	6	7	26
663	9	10	10	10	8	1	12	12	6	9	26
665	9	10	10	10	8	11	12	12	6	7	26
668	9	10	10	10	8	11	12	12	6	7	26
669	9	10	10	10	8	11	12	12	6	9	26

**Table 2:** List of mutations between *rpoB* of WT and that of E9-302.

Codon in WT	Codon in E9-302	Nucleotide (bp)		Deduced amino acid change	

		From	To	From	To
GAG	GGG	424	426	Glutamic acid/E	Glycine/G
AGC	CGC	1525	1527	Serine/S	Arginine/R
CTG	CGG	1531	1533	Leucine/L	Arginine/R

**Table 3:** Comparison of in vitro competitiveness between E9-302 and WT.

Strain	Initial bacteria amount (CFU ml^-1^)	Bacteria amount after incubation (CFU ml^-1^)	*S*
WT	3.23 × 10^8^	6.95 × 10^7^	-0.05
E9-302	2.40 × 10^8^	3.46 × 10^7^	

**Table 4:** LD_50_ of E9-302 and WT.

Dose of challenge (CFU fish^-1^)	Number of death/ Total		Cumulative mortality (%)	

	WT	E9-302	WT	E9-302
1.0 × 10^7^	20/20	17/20	100%	85%
1.0 × 10^6^	20/20	14/20	100%	70%
1.0 × 10^5^	18/20	9/20	90%	45%
1.0 × 10^4^	15/20	6/20	75%	30%
1.0 × 10^3^	11/20	2/20	55%	10%
1.0 × 10^2^	5/20	0/20	25%	0%
LD_50_^a^	8.73 × 10^2^	1.04 × 10^5^	—	—

^a^Zebrafish (length, 3 ± 0.2 cm) were IP injected with WT and E9-302 at different concentration gradient (10^2^ ~ 10^7^ CFU fish^-1^) with three parallel. Data were presented as mean from triplicates. LD_50_ was computed with the Spearman–Kärber method [31] modified from Dias [32].

**Table 5:** RPS results for E9-302 with different vaccination strategies in zebrafish.

Group	Vaccination dose of IP^a^ (CFU fish^-1^)	Cumulative mortality of IP^b^ (%)	RPS of IP^c^ (%)	Primary vaccination dose of IM^d^ (CFU ml^-1^)	Booster vaccination dose of IM^e^ (CFU ml^-1^)	Cumulative mortality of IM^f^ (%)	RPS of IM^g^ (%)
PBS control	N^h^	70	N	N	N	76.7	N
E9-302	1 × 10^3^	10	85.7	4 × 10^7^	2 × 10^7^	26.7	65.2

^a^The fish in each group (20 fish group^-1^) was vaccinated with E9-302 at a dose of 1 × 10^3^ CFU/fish via IP injection.

^b^Zebrafish in the immunized group and control group were challenged with 1 × 10^4^ CFU/fish *E. ictaluri* strain WT at 14 days after IP injection and the cumulative mortality of each group was recorded 12 days after challenge.

^c^Zebrafish in the immunized group and control group were challenged with 1 × 10^4^ CFU/fish *E. ictaluri* strain WT at 14 days after IP injection and the RPS was calculated according to the formula exhibited in 2.7.

^d^The fish in each group (20 fish per group) was immersed in sterile PBS resuspension of E9-302 at a concentration of 4 × 10^7^ CFU/ml for 40 min with continuous aeration, which was considered primary vaccination via immersion (IM).

^e^The fish in each group (20 fish per group) were re-immersed in sterile PBS resuspension of E9-302 at a concentration of 2 × 10^7^ CFU/ml for 40 min with continuous aeration at 14 days after primary IM, which was considered booster vaccination via IM.

^f^Zebrafish in the immunized group and control group were challenged with 1 × 10^4^ CFU/fish *E. ictaluri* strain WT at 7 days after booster IM and the cumulative mortality of each group was recorded 12 days after challenge.

^g^Zebrafish in the immunized group and control group were challenged with 1 × 10^4^ CFU/fish *E. ictaluri* strain WT at 7 days after booster IM and the RPS was calculated according to the formula exhibited in Materials and Methods.

^h^Not assayed or not applicable.

**Table 6:** List of genetic differences between E9-302 and WT.

Gene	Codon in WT	Codon in RE	Nucleotide (bp)^a^		Deduced amino acid change	

			From	To	From	To
*esrB*	CTG	CGG	568	570	**Leucine/L**	**Arginine/R**
*sspH2*	ACG	ACA	1759	1761	Threonine/T	Threonine/T
	GCA	GCG	1765	1767	Alanine/A	Alanine/A
	AGT	AGC	1783	1785	Serine/S	Serine/S
	GTC	ATC	1795	1797	**Valine/V**	**Isoleucine/I**
	CAC	AAC	1825	1827	**Histidine/H**	**Asparagine/N**
	CCG	CAG	1834	1836	**Proline/P**	**Glutamine/Q**
	CTC	TTA	1840	1842	Leucine/L	Leucine/L
	TCA	TCG	1846	1848	Serine/S	Serine/S
	GCG	GTA	1849	1851	**Alanine/A**	**Valine/V**
	GGG	AGT	1852	1854	**Glycine/G**	**Serine/S**
	CTA	TTG	1870	1872	Leucine/L	Leucine/L
	CTA	CTG	1879	1881	Leucine/L	Leucine/L
	GAT	GAG	1882	1884	**Aspartic acid/D**	**Glutamic acid/E**
	CTA	CTG	1885	1887	Leucine/L	Leucine/L
	GTA	ATA	1903	1905	**Valine/V**	**Isoleucine/I**
	AAG	ACG	1909	1911	**Lysine/K**	**Threonine/T**
	TTT	GAT	1918	1920	**Phenylalanine/F**	**Aspartic acid/D**
	−^b^	GA∼AA^b^	-	-	-	**E∼K**
	TTC	GTC	1921	1923	**Phenylalanine/F**	**Valine/V**
	GAA	GAG	1927	1929	Glutamic acid/E	Glutamic acid/E
	GTC	GTT	1936	1938	Valine/V	Valine/V
	CTT	CTG	1942	1944	Leucine/L	Leucine/L
	CAG	CAA	1951	1953	Glutamine/Q	Glutamine/Q
	GAA	AAA	1966	1968	**Glutamic acid/E**	**Lysine/K**
	GAA	GAG	1975	1977	Glutamic acid/E	Glutamic acid/E
	CTA	CTG	1978	1980	Leucine/L	Leucine/L
	ACC	AGC	1981	1983	**Threonine/T**	**Serine/S**
	GGT	GGA	1984	1986	Glycine/G	Glycine/G
	ACT	GCC	1987	1989	**Threonine/T**	**Alanine/A**
	GGA	GAG	1996	1998	**Glycine/G**	**Glutamic acid/E**
	CAA	CGA	2002	2004	**Glutamine/Q**	**Arginine/R**
	GCA	GTA	2014	2016	**Alanine/A**	**Valine/V**
	AGA	CAC	2020	2022	**Arginine/R**	**Histidine/H**
	ACA	ACC	2026	2028	Threonine/T	Threonine/T
	TTA	TCA	2032	2034	**Leucine/L**	**Serine/S**
	AAA	CAA	2041	2043	**Lysine/K**	**Glutamine/Q**
	GCT	GCC	2047	2049	Alanine/A	Alanine/A
	GTG	GTA	2059	2061	Valine/V	Valine/V
	AGT	ACC	2065	2067	**Serine/S**	**Threonine/T**
	CAA	CAG	2080	2082	Glutamine/Q	Glutamine/Q
	AGG	GGG	2086	2088	**Arginine/R**	**Glycine/G**
	GAG	GAA	2107	2109	Glutamic acid/E	Glutamic acid/E
	CCA	CCG	2110	2112	Proline/P	Proline/P
	GGC	AAC	2119	2121	**Glycine/G**	**Asparagine/N**
	AAA	ATA	2128	2130	**Lysine/K**	**Isoleucine/I**
	AGA	AAA	2134	2136	**Arginine/R**	**Lysine/K**
	TCG	GCG	2140	2142	**Serine/S**	**Alanine/A**
	AAA	GAA	2143	2145	**Lysine/K**	**Glutamic acid/E**
	CAG	GAC	2146	2148	**Glutamine/Q**	**Aspartic acid/D**
	CAA	AAA	2152	2154	**Glutamine/Q**	**Lysine/K**
	ACG	GCG	2155	2157	**Threonine/T**	**Alanine/A**
	GAC	AAC	2164	2166	**Aspartic acid/D**	**Asparagine/N**
	ATG	ATT	2173	2175	**Methionine/M**	**Isoleucine/I**
	CAG	GCG	2200	2202	**Glutamine/Q**	**Alanine/A**
	TCT	TAT	2206	2208	**Serine/S**	**Tyrosine/Y**
	CTA	CTG	2218	2220	Leucine/L	Leucine/L
	AAA	GAA	2221	2223	**Lysine/K**	**Glutamic acid/E**
	CTC	GGC	2236	2238	**Leucine/L**	**Glycine/G**
	ATA	CTA	2245	2247	Isoleucine/I	Leucine/L
	GAG	GAA	2254	2256	Glutamic acid/E	Glutamic acid/E
	GGC	GGT	2266	2268	Glycine/G	Glycine/G
	GCT	GCG	2275	2277	Alanine/A	Alanine/A
	GAG	GAA	2290	2292	Glutamic acid/E	Glutamic acid/E
	AAG	AAA	2293	2295	Lysine/K	Lysine/K
	CAC	CGC	2314	2316	**Histidine/H**	**Arginine/R**
	CTA	CTG	2320	2322	Leucine/L	Leucine/L
	TTA	TTG	2332	2334	Leucine/L	Leucine/L
	CTT	CTG	2335	2337	Leucine/L	Leucine/L
	GGC	GCC	2338	2340	**Glycine/G**	**Alanine/A**
	AGG	GGG	2341	2343	**Arginine/R**	**Glycine/G**
Gt39-like	CAG	CA−^c^	700	702	**Glutamine/Q**	Mismatch

^a^The gene differential loci were subject to the loci in the WT sequencing results.

^b^In contrast to WT, there was an insertion of a 27 bp nucleotide sequence. GAGGTCTATGGAGGCGATGAAGATAAA in the *sspH2* gene of the E9-302 sequence, which coded the inferred amino acid sequence EVYGGDEDK.

^c^Compared with WT, E9-302 had a deletion of base G at gene locus 701 in the sequencing of the gene Gt39-like, resulting in a 189 bp sequence mismatch.

The symbol – indicated that there was no corresponding sequence, site information, or inferred amino acid change in the strain WT or E9-302 sequencing results.

The bold letters represented differences in amino acids inferred from different bases.

## References

[1] Klesius PH, Shoemaker CA (1999). Development and Use of Modified Live Edwardsiella ictaluri Vaccine against Enteric Septicemia of Catfish. Advances in Veterinary Medicine.

[2] Zhang Y, Arias CR (2007). Identification and characterization of an intervening sequence within the 23S ribosomal RNA genes of *Edwardsiella ictaluri*. Syst. Appl. Microbiol..

[3] Wagner BA, Wise DJ, Khoo LH, Terhune JS (2002). The epidemiology of bacterial diseases in food-size channel catfish. J. Aquat. Anim. Health.

[4] da Costa A, de Abreu D, Torres Chideroli R, Espirito Santo K, Dib Gonçalves D, Di Santis G (2021). Interspecies transmission of *Edwardsiella ictaluri* in Brazilian catfish (*Pseudoplatystoma corruscans*) from exotic invasive fish species. Dis. Aquat. Organ.

[5] Liu JY, Li AH, Zhou DR, Wen ZR, Ye XP (2010). Isolation and characterization of *Edwardsiella ictaluri* strains as pathogens from diseased yellow catfish *Pelteobagrus fulvidraco* (Richardson) cultured in China: *E. ictaluri* isolates from diseased yellow catfish. Aquac. Res.

[6] Zhou X, Zhang G-R, Ji W, Shi Z-C, Ma X-F, Luo Z-L (2021). The dynamic immune response of yellow catfish (*Pelteobagrus fulvidraco*) infected with *Edwardsiella ictaluri* presenting the inflammation process. Front. Immunol..

[7] Soto E, Griffin M, Arauz M, Riofrio A, Martinez A, Cabrejos ME (2012). *Edwardsiella ictaluri* as the causative agent of mortality in cultured Nile Tilapia. J. Aquat. Anim. Health.

[8] Keskin O, Secer SU, Izgur M, Turkyilmaz S, Mkakosya RS (2004). *Edwardsiella ictaluri* infection in rainbow trout (*Oncorhynchus mykiss*). Turkish J. Vet. Anim. Sci..

[9] Takeuchi H, Hiratsuka M, Hori K, Oinuma H, Umino Y, Nakano D (2021). Environmental factors affecting *Edwardsiella ictaluri* ‐induced mortality of riverine ayu, *Plecoglossus altivelis* (Temminck & Schlegel). J. Fish Dis..

[10] Hawke JP, Kent M, Rogge M, Baumgartner W, Wiles J, Shelley J (2013). Edwardsiellosis caused by *Edwardsiella ictaluri* in laboratory populations of Zebrafish *Danio rerio*. J. Aquat. Anim. Health.

[11] Heuer Ole E, Kruse H, Grave K, Collignon P, Karunasagar I, Angulo Frederick J (2009). Human health consequences of use of antimicrobial agents in aquaculture. Clin. Infect. Dis..

[12] Li J, Mo Z, Li G, Xiao P, Huang J (2015). Generation and evaluation of virulence attenuated mutants of *Edwardsiella tarda* as vaccine candidates to combat edwardsiellosis in flounder (*Paralichthys olivaceus*). Fish Shellfish Immunol..

[13] Goldstein BP (2014). Resistance to rifampicin: a review. J. Antibiot..

[14] Pridgeon JW, Russo R, Shoemaker CA, Klesius PH (2010). Identification of in vitro upregulated genes in a modified live vaccine strain of Edwardsiella ictaluri compared to a virulent parent strain. Comp. Immunol. Microbiol. Infect. Dis..

[15] Olivares-Fuster O, Arias CR (2011). Development and characterization of rifampicin-resistant mutants from high virulent strains of *Flavobacterium columnare*: Rifampicin-resistant mutants of *Flavobacterium columnare*. J. Fish Dis..

[16] Schurig GG, Roop RM, Bagchi T, Boyle S, Buhrman D, Sriranganathan N (1991). Biological properties of RB51; a stable rough strain of *Brucella abortus*. Vet. Microbiol..

[17] Qi Q, Preston GM, MacLean RC (2014). Linking system-wide ompacts of RNA polymerase mutations to the fitness cost of rifampin resistance in *Pseudomonas aeruginosa*. mBio.

[18] LaFrentz BR, LaPatra SE, Call DR, Cain KD (2008). Isolation of rifampicin resistant *Flavobacterium psychrophilum* strains and their potential as live attenuated vaccine candidates. Vaccine.

[19] Sun Y, Liu C-s, Sun L (2010). Isolation and analysis of the vaccine potential of an attenuated *Edwardsiella tarda* strain. Vaccine.

[20] Pridgeon JW, Klesius PH (2011). Development and efficacy of novobiocin and rifampicin-resistant *Aeromonas hydrophila* as novel vaccines in channel catfish and Nile tilapia. Vaccine.

[21] Andersson DI, Hughes D (2010). Antibiotic resistance and its cost: is it possible to reverse resistance?. Nat. Rev. Microbiol..

[22] Hao B, Mo Z, Xiao P, Pan H, Lan X, Li G (2013). Role of alternative sigma factor 54 (RpoN) from *Vibrio anguillarum* M3 in protease secretion, exopolysaccharide production, biofilm formation, and virulence. Appl. Microbiol. Biotechnol..

[23] Leung K, Wong L, Low K, Sin Y (1997). Mini-Tn5 induced growth- and protease-deficient mutants of *Aeromonas hydrophila* as live vaccines for blue gourami, *Trichogaster trichopterus* (Pallas). Aquaculture.

[24] Villain-Guillot P, Bastide L, Gualtieri M, Leonetti J-P (2007). Progress in targeting bacterial transcription. Drug Discov. Today.

[25] Zaw MT, Emran NA, Lin Z (2018). Mutations inside rifampicin-resistance determining region of rpoB gene associated with rifampicin-resistance in Mycobacterium tuberculosis. J. Infect. Public Health.

[26] Kurepina N, Chudaev M, Kreiswirth BN, Nikiforov V, Mustaev A (2022). Mutations compensating for the fitness cost of rifampicin resistance in *Escherichia coli* exert pleiotropic effect on RNA polymerase catalysis. Nucleic Acids Res..

[27] McCallum N, Karauzum H, Getzmann R, Bischoff M, Majcherczyk P, Berger-Bächi B (2006). In vivo survival of teicoplaninresistant *Staphylococcus aureus* and fitness cost of teicoplanin resistance. Antimicrob. Agents Chemother..

[28] Han F, Pu S, Wang F, Meng J, Ge B (2009). Fitness cost of macrolide resistance in *Campylobacter jejuni*. Int. J. Antimicrob. Agents.

[29] Pridgeon JW, Klesius PH (2011). Development and efficacy of a novobiocin-resistant *Streptococcus iniae* as a novel vaccine in Nile tilapia (*Oreochromis niloticus*). Vaccine.

[30] Sander P, Springer B, Prammananan T, Sturmfels A, Kappler M, Pletschette M (2002). Fitness cost of chromosomal drug resistance-conferring mutations. Antimicrob. Agents Chemother..

[31] Hamilton MA, Russo RC, Thurston RV (1977). Trimmed spearman-karber method for estimating median lethal concentrations in toxicity bioassays. Environ. Sci. Technol..

[32] Dias MKR (2016). Lethal dose and clinical signs of *Aeromonas hydrophila* in *Arapaima gigas* (Arapaimidae), the giant fish from Amazon. Vet. Microbiol..

[33] Pridgeon JW, Klesius PH (2011). Development of a novobiocin-resistant *Edwardsiella ictaluri* as a novel vaccine in channel catfish (*Ictalurus punctatus*). Vaccine.

[34] Lin B, Chen S, Cao Z, Lin Y, Mo D, Zhang H (2007). Acute phase response in zebrafish upon *Aeromonas salmonicida* and *Staphylococcus aureus* infection: Striking similarities and obvious differences with mammals. Mol. Immunol..

[35] O'Toole R, von Hofsten J, Rosqvist R, Olsson P-E, Wolf-Watz H (2004). Visualisation of Zebrafish infection by GFP-labelled *Vibrio anguillarum*. Microb. Pathog..

[36] Pressley ME, Phelan PE, Eckhard Witten P, Mellon MT, Kim CH (2005). Pathogenesis and inflammatory response to *Edwardsiella tarda* infection in the zebrafish. Dev. Compar. Immunol..

[37] Hohn C, Lee S-R, Pinchuk LM, Petrie-Hanson L (2009). Zebrafish kidney phagocytes utilize macropinocytosis and Ca^2+^-dependent endocytic mechanisms. PLoS One.

[38] Petrie-Hanson L, Romano CL, Mackey RB, Khosravi P, Hohn CM, Boyle CR (2007). Evaluation of zebrafish *Danio rerio* as a model for Enteric Septicemia of Catfish (ESC). J. Aquat. Anim. Health.

[39] Santander J, Xin W, Yang Z, Curtiss R (2010). The Aspartate-semialdehyde dehydrogenase of *Edwardsiella ictaluri* and its useas balanced-lethal system in fish vaccinology. PLoS One.

[40] Maiden MCJ, Bygraves JA, Feil E, Morelli G, Russell JE, Urwin R (1998). Multilocus sequence typing: a portable approach to the identification of clones within populations of pathogenic microorganisms. Proc. Natl. Acad. Sci. USA.

[41] Maiden MCJ (2006). Multilocus sequence typing of bacteria. Ann. Rev. Microbiol..

[42] Pridgeon JW, Yildirim-Aksoy M, Klesius PH, Srivastava KK, Reddy PG (2012). Attenuation of a virulent *Aeromonas hydrophila* with novobiocin and pathogenic characterization of the novobiocin-resistant strain. J. Appl. Microbiol..

[43] Pridgeon JW, Klesius PH, Yildirim-Aksoy M (2013). Attempt to develop live attenuated bacterial vaccines by selecting resistance to gossypol, proflavine hemisulfate, novobiocin, or ciprofloxacin. Vaccine.

[44] Heckman TI, Shahin K, Henderson EE, Griffin MJ, Soto E (2022). Development and efficacy of *Streptococcus iniae* live-attenuated vaccines in Nile tilapia, *Oreochromis niloticus*. Fish Shellfish Immunol..

[45] Pridgeon JW, Li Y, Yildirim-Aksoy M, Song L, Klesius PH, Srivastava KK (2013). Fitness cost, gyrB mutation, and absence of phosphotransferase system fructose specific IIABC component in novobiocin-resistant *Streptococcus iniae* vaccine strain ISNO. Vet. Microbiol..

[46] Klein JL, Brown TJ, French GL (2001). Rifampin Resistance in *Mycobacterium kansasii* is associated with *rpoB* mutations. Antimicrob. Agents Chemother..

[47] Jin DJ, Gross CA (1988). Mapping and sequencing of mutations in the *Escherichia coli*
*rpoB* gene that lead to rifampicin resistance. J. Mol. Biol..

[48] Choi J, Shin D, Yoon H, Kim J, Lee C-R, Kim M (2010). *Salmonella* pathogenicity island 2 expression negatively controlled by EIIANtr-SsrB interaction is required for Salmonella virulence. Proc. Natl. Acad. Sci.USA.

[49] Tan YP, Zheng J, Tung SL, Rosenshine I, Leung KY (2005). Role of type III secretion in *Edwardsiella tarda* virulence. Microbiology.

[50] Wang X, Wang Q, Xiao J, Liu Q, Wu H, Xu L (2009). *Edwardsiella tarda* T6SS component evpP is regulated by esrB and iron, and plays essential roles in the invasion of fish. Fish Shellfish Immunol..

[51] Liu Y, Zhao L, Yang M, Yin K, Zhou X, Leung KY (2017). Transcriptomic dissection of the horizontally acquired response regulator EsrB reveals its global regulatory roles in the physiological adaptation and activation of T3SS and the cognate effector repertoire in *Edwardsiella piscicida* during infection toward turbot. Virulence.

[52] Otten EG, Werner E, Crespillo-Casado A, Boyle KB, Dharamdasani V, Pathe C (2021). Ubiquitylation of lipopolysaccharide by RNF213 during bacterial infection. Nature.

[53] Xu T, Guo Y, Qi X (2019). Ubiquitination-mediated inflammasome activation during bacterial infection. Int. J. Mol. Sci..

[54] Berglund J, Gjondrekaj R, Verney E, Maupin-Furlow JA, Edelmann MJ (2020). Modification of the host ubiquitome by bacterial enzymes. Microbiol. Res..

[55] Wan M, Wang X, Huang C, Xu D, Wang Z, Zhou Y (2019). A bacterial effector deubiquitinase specifically hydrolyses linear ubiquitin chains to inhibit host inflammatory signalling. Nat. Microbiol..

[56] Hensel M (2000). Salmonella pathogenicity island 2. Mol. Microbiol..

[57] Haraga A, Miller SI (2003). A *Salmonella enterica* serovar typhimurium translocated leucine-rich repeat effector protein inhibits NF-kappa B-dependent gene expression. Infect. Immun..

[58] Kidwai AS, Mushamiri I, Niemann GS, Brown RN, Adkins JN, Heffron F (2013). Diverse secreted effectors are required for *Salmonella* persistence in a mouse infection model. PLoS One.

[59] Halici S, Zenk SF, Jantsch J, Hensel M (2008). Functional analysis of the *Salmonella* pathogenicity island 2-mediated inhibition of antigen presentation in dendritic cells. Infect. Immun..

[60] Bhavsar AP, Brown NF, Stoepel J, Wiermer M, Martin DDO, Hsu KJ (2013). The *Salmonella* type III effector SspH2 specifically exploits the NLR co-chaperone activity of SGT1 to subvert immunity. PLoS Pathog..

[61] Quezada CM, Hicks SW, Galán JE, Stebbins CE (2009). A family of *Salmonella* virulence factors functions as a distinct class of autoregulated E3 ubiquitin ligases. Proc. Natl. Acad. Sci.USA.

[62] Hicks Stuart W, Charron G, Hang Howard C, Galán Jorge E (2011). Subcellular targeting of *Salmonella* virulence proteins by hostmediated S-palmitoylation. Cell Host Microbe.

[63] Wedlich-Soldner R, Li R (2004). Closing the loops: new insights into the role and regulation of actin during cell polarization. Exp. Cell Res..

[64] Walch P, Selkrig J, Knodler LA, Rettel M, Stein F, Fernandez K (2021). Global mapping of *Salmonella enterica*-host proteinprotein interactions during infection. Cell Host Microbe.

[65] Miao EA, Brittnacher M, Haraga A, Jeng RL, Welch MD, Miller SI (2003). *Salmonella* effectors translocated across the vacuolar membrane interact with the actin cytoskeleton: *Salmonella* intracellular effectors. Mol. Microbiol..

[66] Navarro-Garcia F, Serapio-Palacios A, Ugalde-Silva P, Tapia-Pastrana G, Chavez-Dueñas L (2013). Actin cytoskeleton manipulation by effector proteins secreted by diarrheagenic *Escherichia coli* pathotypes. BioMed Res. Int..

[67] Stevens JM, Galyov EE, Stevens MP (2006). Actin-dependent movement of bacterial pathogens. Nat. Rev. Microbiol..

[68] Lu Q, Li S, Shao F (2015). Sweet talk: Protein glycosylation in bacterial interaction with the host. Trends Microbiol..

[69] Tra VN, Dube DH (2014). Glycans in pathogenic bacteria--potential for targeted covalent therapeutics and imaging agents. Chem. Commun. (Camb).

[70] Valguarnera E, Kinsella RL, Feldman MF (2016). Sugar and spice make bacteria not nice: protein glycosylation and its influence in pathogenesis. J. Mol. Biol..

[71] Jia L, Sha S, Yang S, Taj A, Ma Y (2021). Effect of protein O-mannosyltransferase (MSMEG_5447) on *M. smegmatis* and its survival in macrophages. Front. Microbiol..

[72] Liu C-F, Tonini L, Malaga W, Beau M, Stella A, Bouyssié D (2013). Bacterial protein-O-mannosylating enzyme is crucial for virulence of *Mycobacterium tuberculosis*. Proc. Natil. Acad. Sci. USA.

[73] Cain JA, Dale AL, Niewold P, Klare WP, Man L, White MY (2019). Proteomics reveals multiple phenotypes associated with Nlinked glycosylation in *Campylobacter jejuni*. Mol. Cell. Proteomics.

