# Supplementary material for: The Attenuation Mechanism and Live Vaccine Potential of a Low-Virulence Edwardsiella ictaluri Strain Obtained by Rifampicin Passaging Culture
Source: J Microbiol Biotechnol. 2022 Dec 9;33(2):167–79. doi: 10.4014/jmb.2210.10013 (PMC9998210; doi:10.4014/jmb.2210.10013)
Supplement: Supplementary file 1 [file jmb-33-2-167-supple.pdf]

J. Microbiol. Biotechnol.
https://doi.org/10.4014/jmb.2210.10013

Supplementary Materials

Table S1. *E. ictaluri* strains used in the study.

Strain	Source	Geographic origin	Date
656[a]	Yellow catfish	Sichuan Province	2018
658[b]	Channel catfish	Jingzhou City, Hubei Province	2017
663	Yellow catfish	Huzhou City, Zhejiang Province	2007 - 2008
665	Yellow catfish	Huzhou City, Zhejiang Province	2007 - 2008
668	Yellow catfish	Wuhan City, Hubei Province	2018
669	Yellow catfish	Wuhan City, Hubei Province	2018

[a] Strain 656 was donated by Sichuan Agriculture University;

[b] Strain 658 was donated by Yangtze River Fisheries Research Institute of Chinese Academy of Fishery Sciences.

Table S2. Specific PCR primers used for MLST.

Gene	Product length (bp)	Primer sequence (5’ to 3’)
adk	508	*adk*- F: ATTCCGCAGATCTCCAC<br>*adk*- R: TTCACATAGCGAGTATTGC
atpD	884	*atpD*- F: GTTATCGGCGCCGTGGTGGACGT<br>*atpD*- R: TCATCTGCAGGAACGTATACCGC
dnaJ	758	*dnaJ*- F: GATCTGCGTTACAACATGGA<br>*dnaJ*- R: GTCAGATCATCAAAGAATT
gapA	750	*gapA*- F: AACTCACGGTCGTTTCAAC<br>*gapA*- R: CGTTGTCGTACCAAGATAC
glnA	530	*glnA*- F: CCATCGGCGGCTGGAAGGG<br>*glnA*- R: TTGGTCATGGTGTTGAAACG
Y- *hsp60*	565	*Y*- *hsp60*- F: GACGTGGTTGAAGGTATGCA<br>*Y*- *hsp60*- R: CGCCGCCAGCCAGTTTACG
phoR	659	*phoR*- F: GATGGAGTGCTGACTGT<br>*phoR*- R: ATGGCTGACGTTGGCAA
pyrG	722	*pyrG*- F: TGAACTCCGTTGAGTTG<br>*pyrG*- R: TCGTGAACACACCATGT
rpoA	951	*rpoA*- F: ATGCAGGGTTCTGTGACAG<br>*rpoA*- R: GCGGCCAATTTTCAAGGCGC

tuf	884	*tuf*- F: AACATGATCACCGGTGCTGCTCA<br>*tuf*- R: CCAACGGTACGGCCGCCTTCGCG

Table S3 *E. ictaluri* isolates downloaded from MLST database.

Isolate	Country	Year	Host	ST
CECT885	USA	1976	catfish	16
ATCC 33202	USA	1976	*Ictalurus punctatus*	19
NCTC12122	USA	1987	*Ictalurus punctatus*	19
93- 146	USA	1993	catfish	16
S97- 773	USA	1997	*Ictalurus punctatus*	19
S07- 698	USA	2007	*Ictalurus punctatus*	23
RUSVM- 1	USA	2012	*Oreochromis niloticus*	24
2234	Vietnam	2016	*Oreochromis* sp.	24
MS- 17- 156	USA	2017	*Ictalurus punctatus*	19

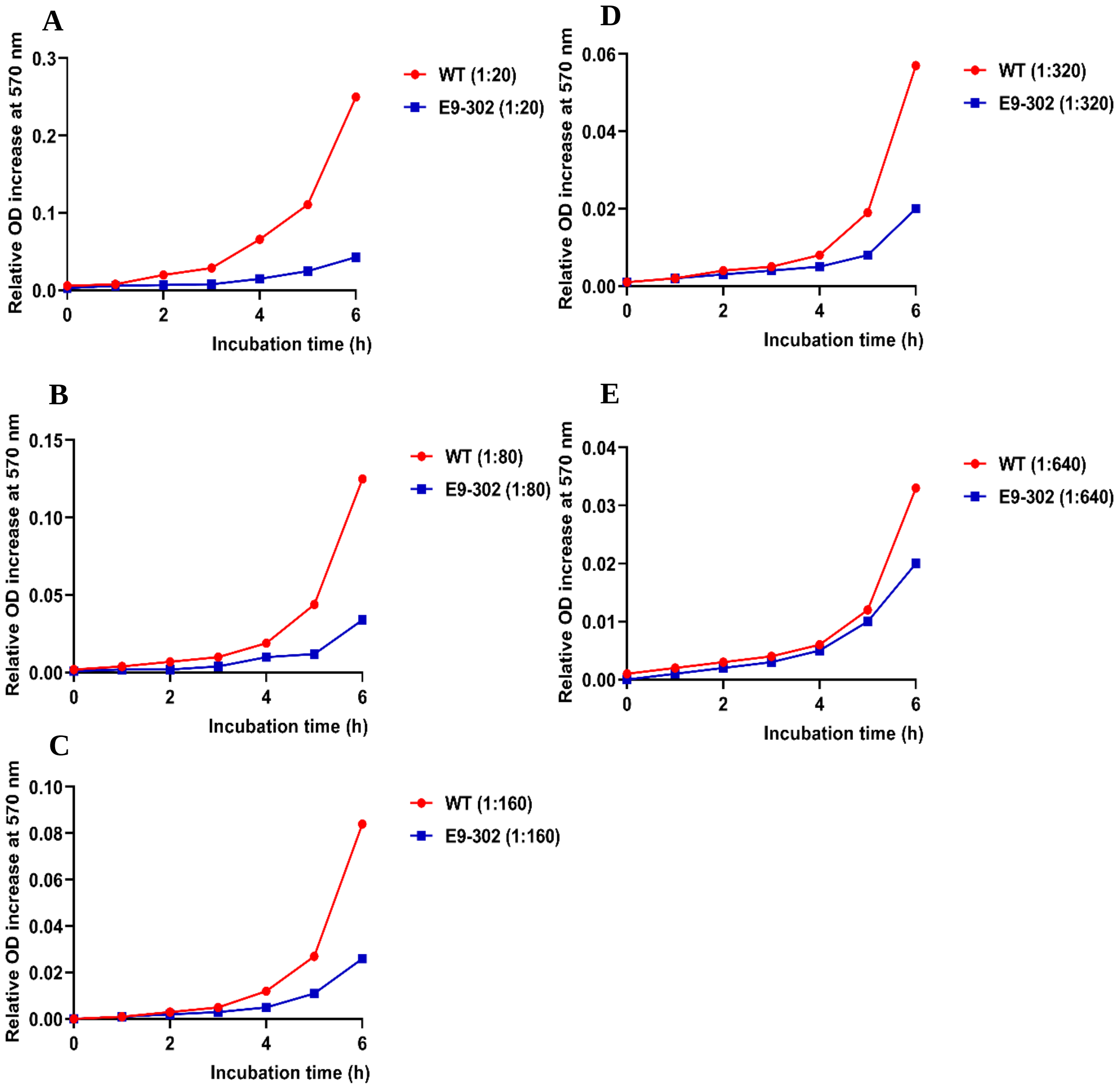

Fig. S1. Cell proliferation rate of *E. ictaluri* E9- 302 compared to that of *E. ictaluri* WT at other dilutions. The number of viable bacteria in each well then determined by dilution method of plate counting.
